# Relationship between selection of dosage forms of vitamin D receptor activators and short-term survival of patients on hemodialysis[Author-notes AUFN1]

**DOI:** 10.1080/0886022X.2021.1995423

**Published:** 2021-11-17

**Authors:** Eri Koshi-Ito, Daijo Inaguma, Shigehisa Koide, Kazuo Takahashi, Hiroki Hayashi, Naotake Tsuboi, Midori Hasegawa, Shoichi Maruyama, Yukio Yuzawa

**Affiliations:** a Department of Nephrology, Nagoya University Graduate School of Medicine, Nagoya, Japan; b Department of Nephrology, Fujita Health University School of Medicine, Toyoake, Japan; c The Aichi Cohort Study of Prognosis in Patients Newly Initiated into Dialysis (AICOPP) Group, Aichi, Japan; d Department of Internal Medicine, Fujita Health University Bantane Hospital, Nagoya, Japan

**Keywords:** Vitamin D, dialysis, mortality, vitamin D receptor activator, chronic kidney disease

## Abstract

**Background:**

The benefits of vitamin D receptor activators (VDRAs) for patients with chronic kidney disease are well recognized. However, the optimal criteria for patient selection, dosage forms, and duration providing the highest benefit and the least potential risk remain to be confirmed.

**Materials and methods:**

The study population was derived from the Aichi Cohort Study of Prognosis in Patients Newly Initiated into Dialysis, a multicenter prospective cohort study of 1520 incident dialysis patients. According to the VDRA usage status in March 2015 (interim report), the 967 patients surviving after March 2015 were classified into three groups: without VDRA (NV, *n* = 177), oral VDRA (OV, *n* = 447), and intravenous VDRA (IV, *n* = 343). Mortality rates were compared using the log-rank test, and factors contributing to all-cause mortality were examined using both univariate and multivariate Cox proportional hazard regression analyses.

**Results:**

There were 104 deaths (NV, *n* = 27; OV, *n* = 53; IV, *n* = 24) during the follow-up period (1360 days, median), and significant differences in cumulative survival rates were observed between the three groups (*p* = 0.010). Moreover, lower all-cause mortality was associated with IV versus NV (hazard ratio, 0.46 [95% confidence interval 0.24–0.89]; *p* = 0.020).

**Conclusion:**

This study demonstrated the impact of the VDRA dosage form on the short-term survival of incident hemodialysis patients during the introduction period. Our results suggest that relatively early initiation of intravenous VDRA in patients beginning hemodialysis may have some clinical potential.

## Introduction

The kidneys play an important role in regulating mineral metabolism. In chronic kidney disease (CKD), imbalance of minerals leads not only to functional abnormality of bones and parathyroid glands but also to a systemic complication known as chronic kidney disease-mineral and bone disorder (CKD-MBD). This condition is strongly associated with mortality.

The treatment strategy for CKD-MBD includes dietary phosphorus restriction, phosphate binders, vitamin D receptor activators (VDRAs), and calcimimetics. Among these, VDRAs play a central role as medications for individuals with CKD-MBD. Many studies have reported that vitamin D status is related to survival and that supplementation with native vitamin D or administration of VDRA contributes to better prognosis [1–5]. The benefits are believed to be derived not only from an improvement in the balance of calcium and phosphorus metabolism and aiding in the prevention of osteoporosis [6], but also from pleiotropic effects on responsiveness to erythropoiesis-stimulating agents [7], left ventricular hypertrophy [8–11], insulin resistance [12], and the immune system [13]. However, there is a possibility that VDRAs may lead to ectopic calcification, especially vascular calcification, through hypercalcemia and hyperphosphatemia [14].

Serum calcitriol levels are known to decrease after the early stages of CKD. A previous report [15] demonstrated that in 1,814 CKD patients in the United States, serum calcitriol levels (defined as vitamin D) were below 22 pg/mL in 13% of the patients whose estimated glomerular filtration rate (eGFR) was ≥80 mL/min/1.73 m^2^ and more than 60% of the patients whose eGFR was <30 mL/min/1.73 m^2^. Various mechanisms have been proposed for this result [16–18]. Hyperphosphatemia, shedding of proximal tubule cells, and increased levels of fibroblast growth factor 23 (FGF23) lower the expression level of 1α-hydroxylase, which converts 25-hydroxyvitamin D into calcitriol. Furthermore, a reduction in the eGFR and loss of megalin causes a decrease in the amount of 25-hydroxyvitamin D reaching the renal tubules that promote its reabsorption. Alleviation of calcitriol deficiency by VDRAs is most likely associated with the benefits mentioned above.

According to clinical practice guidelines for the management of CKD-MBD from the Japanese Society for Dialysis Therapy (JSDT), dietary phosphorus restriction and/or the use of calcium-containing phosphorus binders and oral VDRA are recommended for controlling parathyroid hormone (PTH) levels in pre-dialysis patients. Meanwhile, serum phosphorus/calcium management and VDRA and/or cinacalcet hydrochloride is recommended for decreasing the levels of intact PTH in dialysis patients [19]. Recently, the Kidney Disease Improving Global Outcomes (KDIGO) 2017 clinical practice guidelines suggested that calcitriol and vitamin D analogs should not be used routinely for adult patients with CKD G3a–G5 who are not on dialysis, and it is reasonable to limit the use of calcitriol and vitamin D analogs for patients with CKD G4–G5 who suffer from severe and progressive hyperparathyroidism [20]. Therefore, the optimal time to begin or discontinue VDRA therapy, the type of VDRA to be administered, and the optimal route of administration (i.e., intravenous or oral) to maximize survival in CKD patients remains unclear.

The current study is a posthoc analysis using data derived from the Aichi Cohort Study of Prognosis in Patients Newly Initiated into Dialysis (AICOPP). The AICOPP defined laboratory and physical data collected prior to the first session of hemodialysis as baseline data and documented patient survival. The AICOPP collected information on the type of VDRA that the patients were administered at baseline, and on 1st March 2015. One of the unique points of this study was that patient survival was observed in three consecutive phases: the end stage of kidney disease just before introducing hemodialysis, the introduction period of hemodialysis, and the maintenance dialysis period (<3.5 years). According to an investigation by the JSDT, in 2015 the patient survival rate within 5 years after dialysis initiation was not higher than 59.8% [21]. Therefore, we examined the impact of the VDRA dosage form on the short-term survival of incident hemodialysis patients during the hemodialysis introduction period.

## Materials and methods

### Subjects

**Figure 1.**
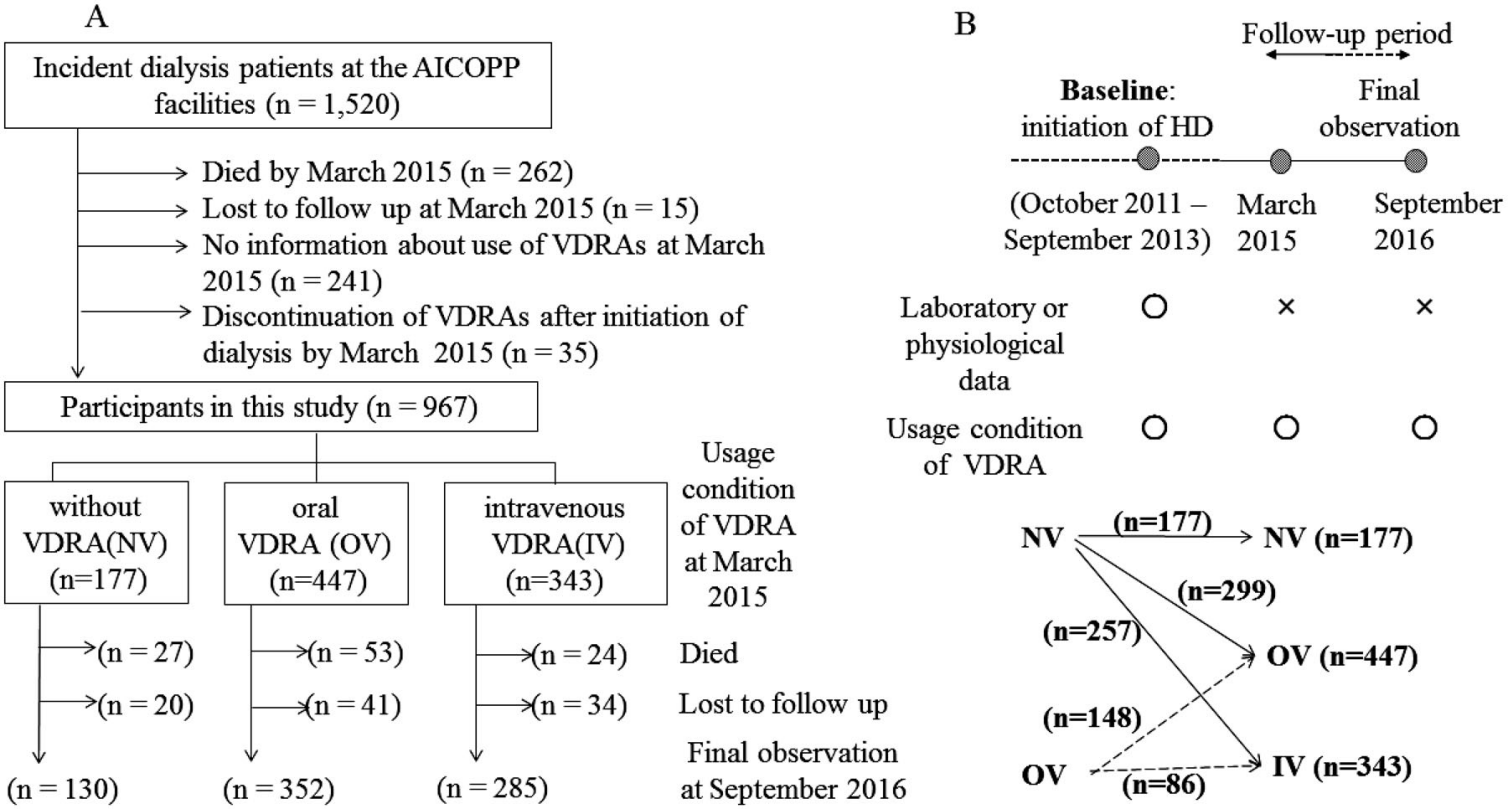
Participated patients’ flowchart. (A) Definitions of the three main groups and details of follow-up. (B) Definitions of baseline and follow-up period, and changes of VDRA usage status from the time of initiation of dialysis (baseline) to March 2015 (interim report). IV: intravenous VDRA group; OV: oral VDRA group; NV: without VDRA group.

The study population was derived from the AICOPP, multicenter prospective cohort analysis of 1520 patients who began dialysis at one of the 17 centers involved between October 2011 and September 2013 [22], and who were followed until September 2016 (study registration number UMIN000007096). The patient flowchart is presented in Figure 1(A). Since it was necessary to know the patient’s vitamin D usage status as of March 2015 (interim report), in addition to the baseline (as described below), patients who survived after March 2015 were targeted. Of 1520 patients, we excluded 262 who died in March of 2015, 15 who were lost to follow-up, 241 with unconfirmed information regarding the use of VDRA in March 2015, and 35 who discontinued VDRA between dialysis initiation and March 2015. Ultimately, 967 patients were enrolled in the present study. The follow-up period was defined as the survival period after initiation of dialysis.

### Patient characteristics and data at the time of dialysis initiation (baseline)

Baseline was defined as the time of dialysis initiation. The patient’s body mass index (BMI) was measured at the first dialysis session, and cardiovascular (CV) history and history of malignancy were obtained from medical records and categorized as coronary artery disease, valvular heart disease, congestive heart failure requiring hospitalization, cerebral infarction, cerebral hemorrhage, or aortic disease. Diabetes comorbidity was defined as a fasting blood glucose level ≥126 mg/dL, random blood glucose level ≥200 mg/dL, glycated hemoglobin A1c level (National Glycohemoglobin Standardization Program) ≥6.5%, insulin use, or use of oral hypoglycemic agents. Before the first dialysis session, blood samples were drawn for laboratory investigations, and patient blood pressure was measured. Aortic calcification was assessed according to the presence of aortic arch calcification on plain frontal chest radiographs captured immediately before dialysis initiation. Cardiac valve calcification was assessed according to the presence of a calcified aortic or mitral valve determined by B-mode echocardiography during the 1-month period before and after dialysis initiation. The eGFR was calculated using the Modification of diet in renal disease (MDRD) study equation, with a coefficient specifically modified for Japanese people, as advocated by the Japanese Society of Nephrology [23]. If serum calcium was less than 4 mg/dL, adjusted calcium was calculated using Payne’s equation [24], and if not, the original serum calcium itself was regarded as ‘adjusted calcium’. In addition, information regarding medication use was obtained from patient medical records. VDRAs and angiotensin-converting enzyme inhibitors (ACEIs)/angiotensin receptor blockers (ARBs) were considered medications at the time of dialysis initiation if they had been used for at least 3 months previously. However, other medications were referred to as drugs taken by the patients at the time of dialysis initiation.

### Classification according to the use of VDRA

Prescriptions for VDRA were surveyed not only at baseline but also on 1st March 2015 as an interim report (Figure 1(B)). Data were acquired from medical records or questionnaires obtained from other institutions. Patients were classified into three groups according to VDRA use in March 2015: no use of VDRA (NV), oral VDRA (OV), and intravenous VDRA (IV). Outcomes were compared between the three groups. The OV group was administered oral calcitriol and alpha-calcidol, whereas the IV group was administered intravenous calcitriol and maxacalcitol. Paricalcitol, which has been reported to confer survival benefits in dialysis patients [25], was not available in Japan.

### Outcomes

Survival on 30th September 2016, was confirmed from medical records. Information was obtained through a mail survey for patients transferred to other institutions. The starting point for the survival time was defined as March 2015. Five outcomes were defined and compared between the three groups: all-cause mortality, CV, infection, cancer, and non-cancer-related mortality. CV death was defined as death caused by heart failure, acute coronary syndrome, stroke, or cardiogenic sudden death.

### Subgroup analysis

Mortality was compared between the OV and IV groups, which included patients who had already undergone VDRA treatment at dialysis initiation. Moreover, the three groups were subdivided into five subgroups based on the VDRA usage form at the initiation of dialysis: Subgroup a, which did not use VDRA at both points (NV-NV); Subgroup b, which used oral VDRA in March 2015 but not at dialysis initiation (NV-OV); Subgroup c, which used oral VDRA at both points (OV-OV); Subgroup d, which had not used any forms of VDRA at the initiation of dialysis but used intravenous VDRA in March 2015 (NV-IV); and Subgroup e, which had used oral VDRA at the initiation of dialysis but used intravenous VDRA in March 2015 (OV-IV). The all-cause mortality hazard ratio of each subgroup was compared to Subgroup a in the three adjusted models described below.

### Statistical analysis

Statistical analysis was performed using SPSS version 24 (IBM Corporation, Armonk, NY, USA) and the Easy R program (Saitama Medical Center, Jichi Medical University, Saitama, Japan) [26]. Patient characteristics and baseline data were compared between the three groups by the Kruskal-Wallis test for continuous variables and Fisher’s exact test for nominal variables. The significance of differences in pairwise comparisons was examined by posthoc analysis of the Steel–Dwass test for continuous variables and Bonferroni’s multiple comparisons. Mortality rates were compared between the three groups using the log-rank test on Kaplan–Meier curves. Factors contributing to all-cause mortality were examined using univariate Cox proportional hazards regression analysis. In addition to the three groups, factors that were significant in the univariate analysis served as explanatory variables for the multivariate Cox proportional hazard analysis using the stepwise method (i.e., diabetes comorbidity, history of cardiovascular disease (CVD), BMI, diastolic blood pressure, aortic calcification, eGFR, serum phosphate, serum magnesium, and use of ACEIs/ARBs). In addition, we tested all-cause mortality in the five subgroups, constructing models and performing Cox proportional hazard analysis. For stratified analyses, all-cause mortality rates were compared using univariate Cox proportional hazard models as follows: Model 1 (adjusted for age and sex); Model 2 (adjusted for Model 1 plus diabetes comorbidity, history of CVD, BMI, diastolic blood pressure, aortic calcification, eGFR, serum phosphate, serum magnesium, and use of ACEIs/ARBs); and Model 3 (adjusted for Model 2 plus serum-adjusted calcium). Continuous variables are expressed as mean ± standard deviation or as median (interquartile range), and categorical variables are presented as percentages. Differences were considered statistically significant at *p* < 0.05.

## Results

### Comparison of patient characteristics and baseline data among the three primary groups

Patient characteristics and baseline data are summarized in Table 1. The median follow-up period from initiation of dialysis was 1360 days, with no significant differences (*p* = 0.951) between the three primary groups (i.e., NV, OV, and IV). However, there was non-uniformity among the three groups about some parameters. Post-hoc analysis for pairwise comparison revealed that the IV group showed significantly lower diabetes comorbidity, serum adjusted calcium, magnesium, and bicarbonate, and higher serum alkaline phosphatase and intact PTH compared with the remaining two groups. The analysis also demonstrated that there were significantly more females in the NV group than the OV group, and hemoglobin in the OV group was higher than in the IV group. The difference in the use of phosphate binders was not significant in the pairwise comparison. The most common causes of renal failure were diabetic nephropathy (45.8%), nephrosclerosis (24.1%), and chronic glomerulonephritis (14.0%). The pairwise comparison demonstrated that the proportion of diabetic nephropathy was significantly lower in the IV group than in the other groups.

**Table 1.** Baseline characteristics and laboratory data before commencing dialysis.

Variables	Without VDRAs (NV, *n* = 177)	Oral VDRAs (OV, *n* = 447)	Intravenous VDRAs (IV, *n* = 343)	*p* value
Age (years old)	70 [58–76]	68 [60–76]	69 [60–78]	0.472
Female Gender (%)	42.9	31.1	35.9	0.018*
Diabetes Mellitus (%)	63.3	58.8	48.1	0.001*
History of CAD (%)	13.0	15.2	13.5	0.680
History of Stroke (%)	15.8	16.1	12.5	0.342
History of CVD (%)	42.4	43.8	37.9	0.234
History of Malignancy (%)	8.5	8.5	8.5	1
BMI (kg/m^2^)	23.0 [20.7–26.1]	23.1 [20.8–25.7]	23.5 [21.3–26.3]	0.248
SBP (mmHg)	155 ± 29	154 ± 25	152 ± 26	0.446
DBP (mmHg)	76 [66–88]	78 [68–88]	78 [68–88]	0.566
Aortic Calc (%)	37.5	33.8	36.3	0.618
Aortic Valve Calc (%)	31.8	28.2	30.3	0.672
Period from dialysis initiation to final observation (days)	1386 [1219–1583]	1352 [1218–1571]	1373 [1217–1569]	0.951
Period from initiation of dialysis to 1st March 2015 (days)	859 [719–1058]	846 [699–1045]	851 [691–1047]	0.656
Follow-up period (Period from 1st March 2015 to final observation) (days)	579 [530–579]	579 [579–579]	579 [579–579]	0.0641
Laboratory data				
Hemoglobin (g/dL)	8.9 [8.1–9.4]	9.5 [8.6–10.5]	9.3 [8.2–10.2]	0.0309*
Albumin (g/dL)	3.1 [2.7–3.5]	3.2 [2.8–3.6]	3.3 [2.9–3.6]	0.0915
ALP (IU/L)	215 [173–268]	225 [178–279]	249 [201–319]	<0.001*
Uric Acid (mg/dL)	8.5 [7.6–10.3]	8.5 [7.0–9.8]	8.4 [7.1–10.0]	0.202
BUN (mg/dL)	86.1 [73.0–107.0]	88.0 [73.7–102.6]	87.0 [69.6–107.0]	0.912
Creatinine (mg/dL)	8.45 [7.08–10.0]	8.64 [7.28–10.5]	8.71 [7.21–10.2]	0.372
eGFR (mL/min/1.73m^2^)	5.0 [4.0–6.0]	4.95 [4.08–6.08]	4.82 [3.89–6.03]	0.509
Adjusted Calcium (mg/dL)	8.9 [8.1–9.4]	8.8 [8.0–9.3]	8.6 [7.8–9.1]	<0.001*
Phosphate (mg/dL)	6.4 [5.3–7.5]	6.2 [5.1–7.1]	6.1 [5.2–7.1]	0.218
Magnesium (mg/dL)	2.2 [1.9–2.5]	2.2 [1.9–2.4]	2.0 [1.8–2.3]	<0.001*
LDL-C (mg/dL)	86 [66–108]	84 [67–107]	84 [65–109]	0.620
HDL-C (mg/dL)	43 [33–55]	43 [33–54]	42 [33–52]	0.625
TG (mg/dL)	111 [86–148]	113 [83–153]	110 [78–149]	0.402
Ferritin (ng/mL)	131 [76–272]	122 [63–207]	121 [59–203]	0.104
Intact PTH (pg/mL)	244 [158–335]	260 [186–381]	407 [281–591]	<0.001*
1,25(OH)_2_ vitamin D (pg/mL)	10.1 [7.0–13.7]	12.6 [8.0–17.0]	12.3 [8.2–19.0]	0.0073*
Bicarbonate (mmol/L)	19.4 [17.5–22.6]	19.7 [16.9–22.3]	18.6 [15.9–21.8]	0.0116*
CRP (mg/dL)	0.31 [0.10–1.43]	0.26 [0.10–1.14]	0.24 [0.11–1.09]	0.461
Medication				
ACEIs / ARBs (%)	67.8	61.0	60.3	0.211
CCBs (%)	74.6	81.2	83.4	0.052
Loop diuretics (%)	67.2	66.9	65.3	0.866
βBs (%)	31.6	33.8	33.5	0.872
Statins (%)	36.7	40.5	41.1	0.603
VDRAs (%)	0.0	33.1	25.1	<0.001*
Phosphate binders (%)	33.9	42.1	34.1	0.037*
NaHCO_3_ (%)	42.9	46.5	46.9	0.657
ESAs (%)	85.3	87.2	88.9	0.496
Cause of renal failure				
Diabetic nephropathy (%)	53.7	49.4	37.0	<0.001*
Nephrosclerosis (%)	22.0	20.1	30.3	0.0035*
Chronic glomerulonephritis (%)	8.47	13.0	18.1	0.0078*

Data: Mean ± standard deviation; Median [1st quartile to 3rd quartile].

Significant *p-*values are marked with ‘*’.

VDRA: vitamin D receptor activator, IV: intravenous, CAD: coronary artery disease, CVD: cardiovascular disease, BMI: body mass index, SBP: systolic blood pressure, DBP: diastolic blood pressure, Calc: calcification, ALP: alkaline phosphatase, BUN: blood urea nitrogen, eGFR: estimated glomerular filtration rate, LDL-C: low density lipoprotein cholesterol, HDL-C: high density lipoprotein cholesterol, TG: triglyceride, PTH: parathyroid hormone, CRP: C reactive protein, ACEI: angiotensin converting enzyme, ARB: angiotensin receptor blocker, CCB, calcium channel blocker, βB: β blocker, VDRA: vitamin D receptor activator, ESA: erythropoiesis stimulating agent.

### Comparison of all-cause mortality

**Figure 2.**
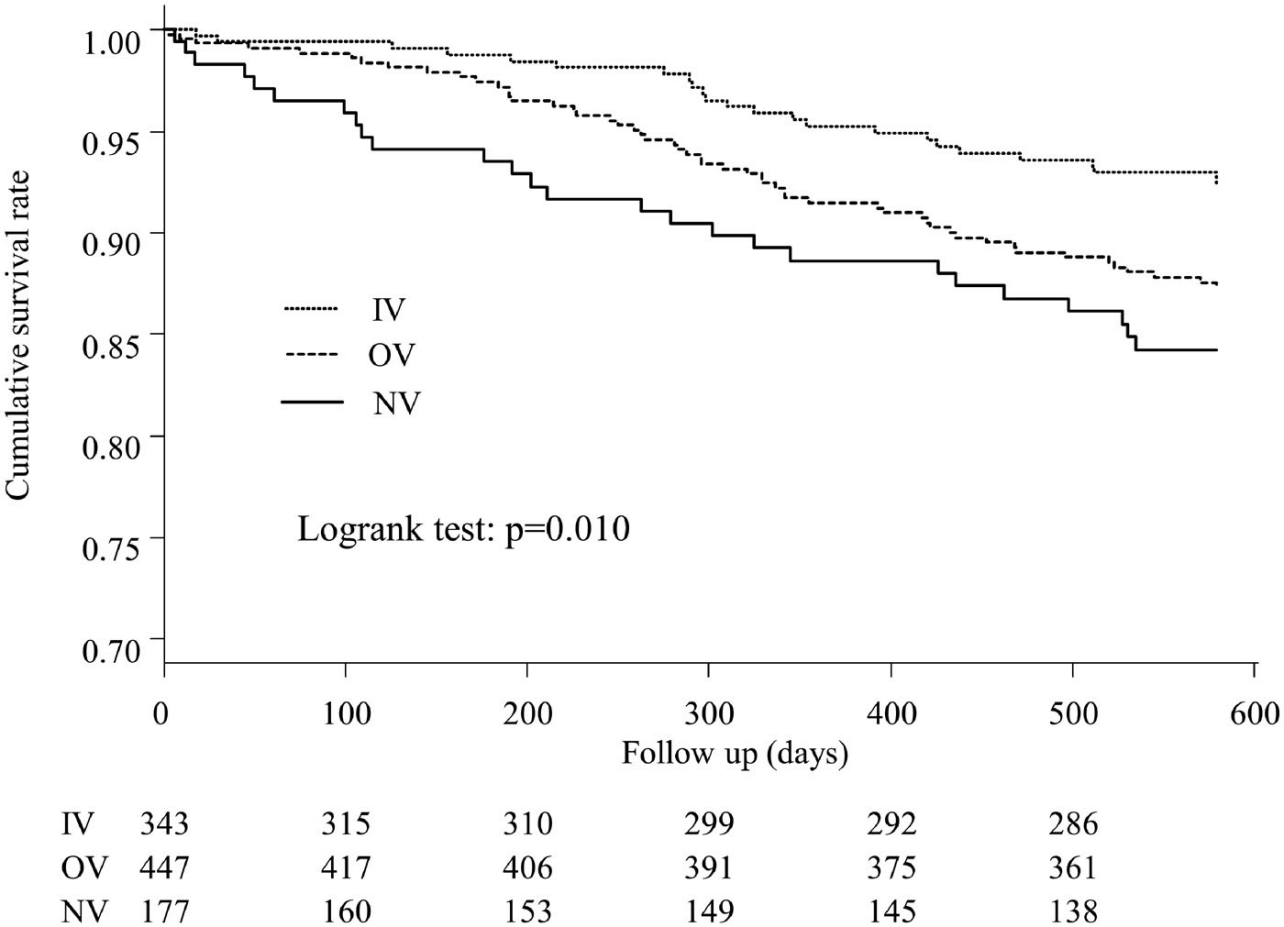
Kaplan–Meier curves for the cumulative survival between the three groups. Significant differences between the cumulative survival rates were observed for the three groups (*p* = 0.010). IV: intravenous VDRA group; OV: oral VDRA group; NV: without VDRA group.

Kaplan–Meier curves for cumulative survival rates in the three groups are shown in Figure 2. There were 104 deaths among the three groups during the follow-up period (NV, *n* = 27; OV, *n* = 53; IV, *n* = 24). Significant differences in cumulative survival rates were observed between the three groups (*p* = 0.010). There was no significant difference in mortality between the two patient groups who had already taken VDRAs at dialysis initiation (*p* = 0.058) (Supplementary Figure 1).

### Comparison of CV-, infection-, cancer-, and non-cancer-related mortality

**Figure 3.**
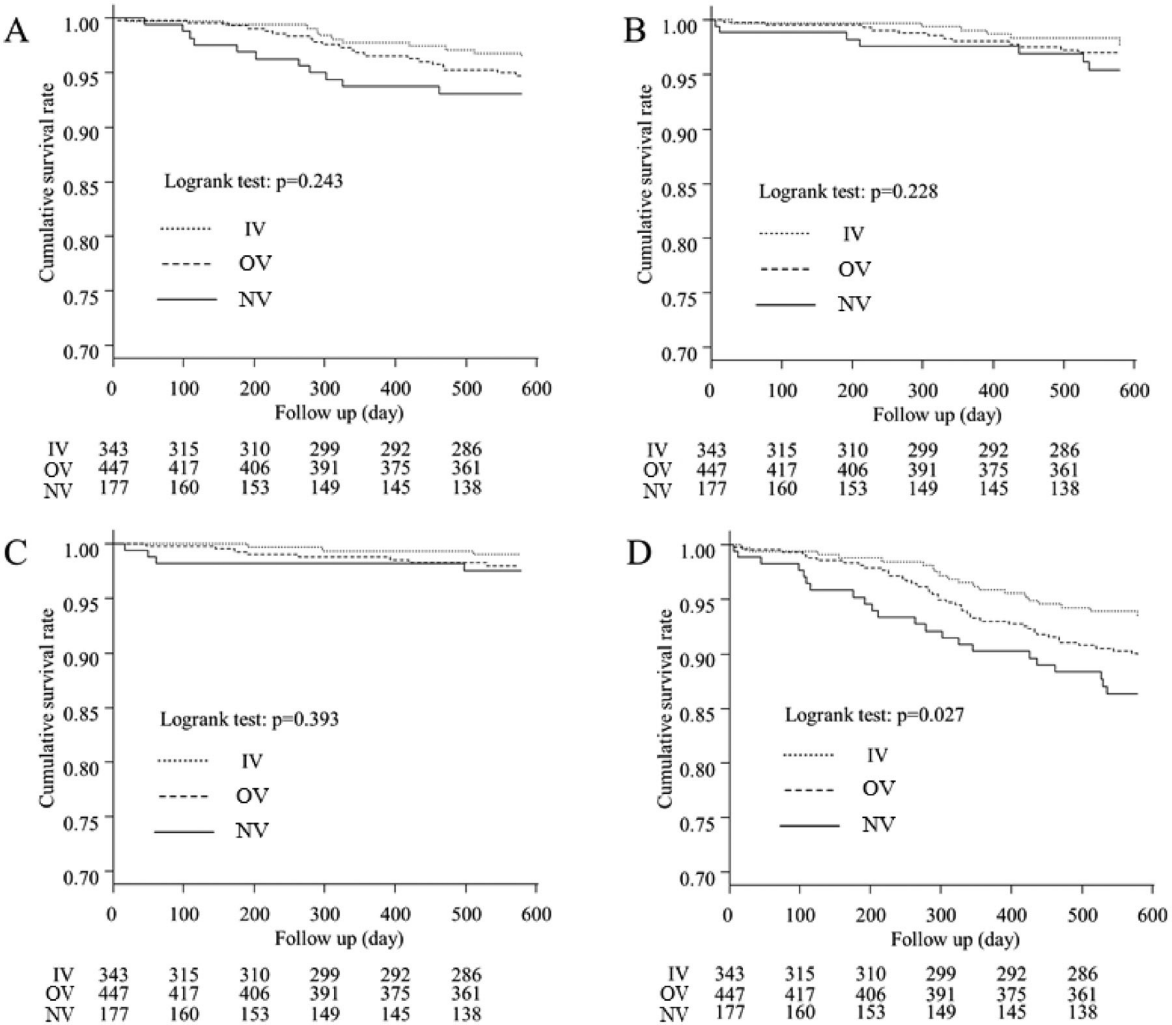
Kaplan–Meier curves for the cumulative survival for each cause of death between the three groups. (A) Comparison of mortality from cardiovascular diseases, (B) Comparison of mortality from infection, (C) Comparison of mortality from cancer, (D) Comparison of mortality from non-cancer causes. Significant differences between the non-cancer-related mortality were observed for the three groups (*p* = 0.027). IV: intravenous VDRA group; OV: oral VDRA group; NV: without VDRA group.

Kaplan–Meier curves for the three groups showing CV-, infection-, cancer-, and non-cancer-related mortality are presented in Figure 3(A–D), respectively. There were significant differences in non-cancer-related mortality between the three groups (*p* = 0.027). However, there were no significant differences in CV-, infection-, and cancer-related mortality (*p* = 0.243, *p* = 0.228, and *p* = 0.393, respectively).

### Hazard ratios for all-cause mortality among the three groups

The univariate regression analyses (Table 2) demonstrated that all-cause mortality rates were significantly lower in the IV group than those in the NV group (hazard ratio (HR) 0.44 [95% confidence interval (CI) 0.25–0.76]; *p* = 0.003), but not significantly different from those in the OV group (HR 0.75 [95% CI 0.47–1.19]; *p* = 0.225). In addition, all-cause mortality was associated with various factors including age, sex, history of CVD, BMI, diastolic blood pressure, aortic calcification, eGFR, serum calcium, serum phosphate, serum magnesium, and use of ACEIs/ARBs. The results of the multivariate Cox proportional hazard analysis using the stepwise method are shown in Table 3. The IV group all-cause mortality rate was lower than that of the NV group (HR, 0.46;95% CI 0.24–0.89; *p* = 0.02). In addition, higher mortality was associated with older age, male sex, and increased serum calcium levels.

**Table 2.** Associations of variables with all-cause mortality according to the univariate Cox proportional hazard regression analysis.

Variables	Hazard ratio	95% CI	*p*-Value
IV vs NV	0.44	0.25–0.76	0.003
OV vs NV	0.75	0.47–1.19	0.225
Age (10 years old)	2.06	1.68–2.53	<0.001
Female Gender	0.54	0.34–0.85	0.005
Diabetes Mellitus	1.12	0.76–1.66	0.560
History of CAD	1.46	0.90–2.38	0.129
History of Stroke	1.59	0.99–2.54	0.054
History of CVD	2.07	1.40–3.05	<0.001
BMI (1 kg/m^2^)	0.93	0.88–0.98	0.005
SBP (10 mmHg)	0.99	0.93–1.06	0.801
DBP (10 mmHg)	0.90	0.91–1.00	0.050
Aortic Calc	1.87	1.28–2.75	0.001
Aortic Valve Calc	1.39	0.90–2.16	0.143
Hemoglobin (1 g/dL)	1.10	0.97–1.26	0.131
Albumin (1 g/dL)	0.82	0.60–1.12	0.215
ALP (10 IU/L)	1.01	0.99–1.02	0.289
Uric Acid (mg/dL)	1.00	0.93–1.09	0.945
BUN (10 mg/dL)	1.02	0.96–1.09	0.476
Creatinine (1 mg/dL)	0.86	0.80–0.93	<0.001
eGFR (1 mL/min/1.73m^2^)	1.09	1.02–1.16	0.010
Adjusted Calcium (1 mg/dL)	1.38	1.14–1.67	0.001
Phosphate (1 mg/dL)	0.83	0.74–0.94	0.003
Magnesium (1 mg/dL)	1.53	1.07–2.19	0.020
LDL-C (10 mg/dL)	0.96	0.90–1.03	0.220
HDL-C (10 mg/dL)	1.11	0.98–1.24	0.101
TG (10 mg/dL)	1.01	0.98–1.03	0.538
Ferritin* (1 ng/mL)	1.00	0.99–1.00	0.917
Intact PTH* (1 pg/mL)	1.00	0.99–1.00	0.180
Bicarbonate (1 mmol/L)	1.03	0.99–1.08	0.193
CRP* (1 mg/dL)	1.03	0.98–1.07	0.229
ACEIs/ARBs	0.64	0.44–0.94	0.023
CCBs	1.09	0.65–1.80	0.754
Loop diuretics	1.15	0.76–1.75	0.503
βBs	1.00	0.66–1.50	0.989
Statins	0.72	0.47–1.08	0.108
Phosphate binders	0.70	0.46–1.07	0.096
NaHCO_3_	0.97	0.66–1.43	0.882
ESAs	0.96	0.54–1.72	0.901

CI: confidence interval; IV: intravenous VDRA (vitamin D receptor activator) group; OV: oral VDRA group; NV: without VDRA group; CAD: coronary artery disease; CVD: cardiovascular disease; BMI: body mass index; SBP: systolic blood pressure; DBP: diastolic blood pressure; Calc: calcification; ALP: alkaline phosphatase; BUN: blood urea nitrogen; eGFR: estimated glomerular filtration rate; LDL-C: low density lipoprotein cholesterol; HDL-C: high density lipoprotein cholesterol; TG: triglyceride; PTH: parathyroid hormone; CRP: C reactive protein; ACEI: angiotensin converting enzyme; ARB: angiotensin receptor blocker; CCB: calcium channel blocker; βB: β blocker; ESA: erythropoiesis stimulating agent.

**Table 3.** Associations of variables with all-cause mortality according to the multivariate Cox proportional hazard regression analysis.

Variables	Hazard ratio	95%CI	*p*-Value
IV vs NV	0.46	0.24–0.89	0.022
Age (10 years old)	2.04	1.56–2.67	<0.001
Female gender	0.42	0.23–0.77	0.005
Adjusted calcium (1 mg/dL)	1.49	1.13–1.96	0.005

CI: confidence interval; IV: intravenous VDRA (vitamin D receptor activator) group; NV: without VDRA group.

Adjusted for DM: history of CVD: BMI: DBP: aortic calcification: eGFR: serum phosphate: serum magnesium: and use of ACEIs/ARBs.

DM: diabetes mellitus; CVD: cardiovascular disease; BMI: body mass index; DBP: diastolic blood pressure; eGFR: estimated glomerular filtration rate; ACEI: angiotensin converting enzyme inhibitor; ARB: angiotensin receptor blocker.

### Comparison of all-cause mortality and HRs among the five subgroups

**Figure 4.**
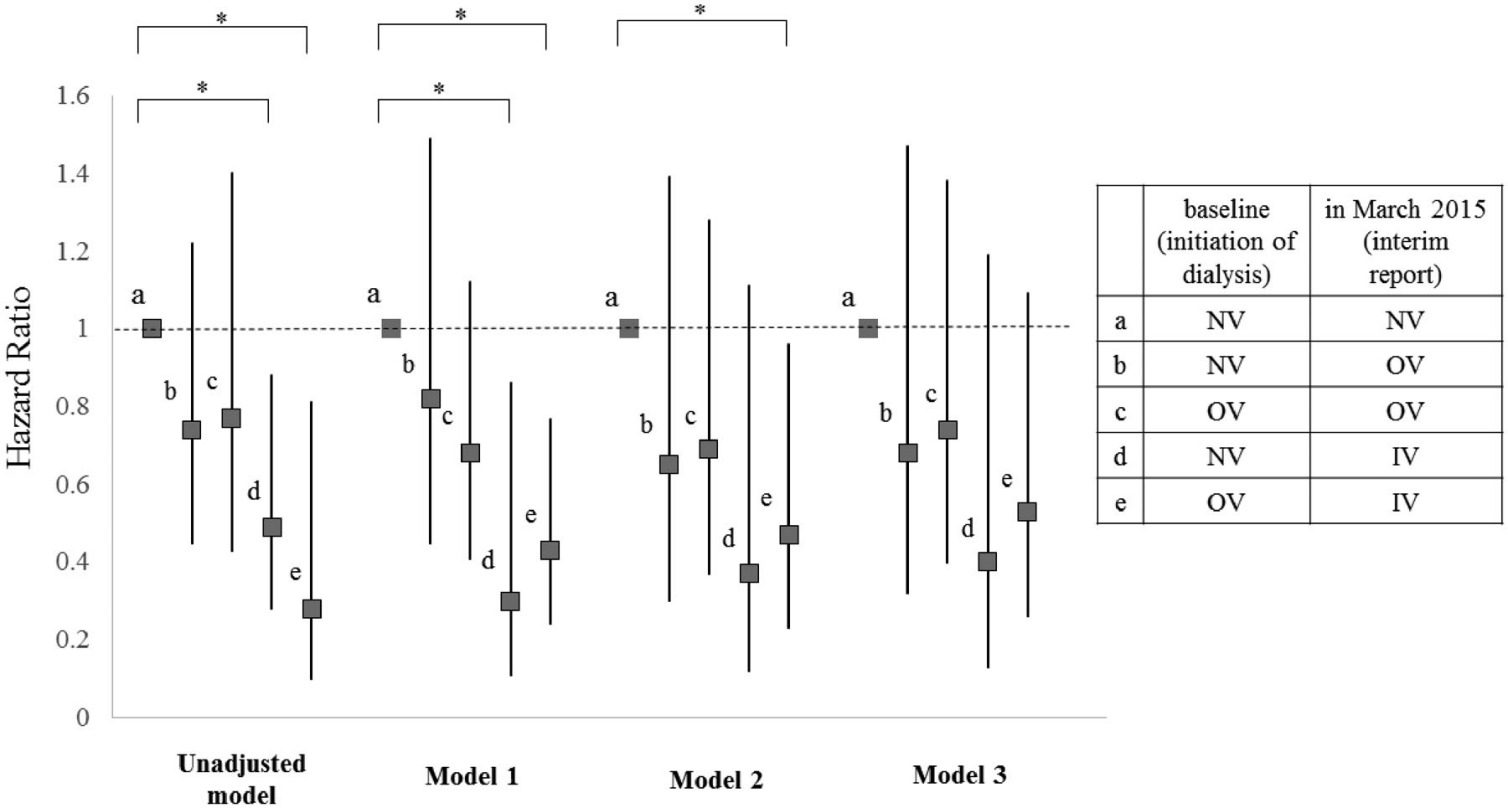
Hazard ratio for all-cause mortality of each subgroup compared to Subgroup 1. Subjects were subdivided into five subgroups according to which form of VDRA was used at both of two points: initiation of dialysis (at baseline) and March 2015 (at interim report). Subgroup a, which did not use VDRA at both points (NV-NV); Subgroup b, which used oral VDRA in March 2015 but not at dialysis initiation (NV-OV); Subgroup c, which used oral VDRA at both points (OV-OV); Subgroup d, which had not used any forms of VDRA at initiation of dialysis but used intravenous VDRA in March 2015 (NV-IV); and Subgroup e, which had used oral VDRA at initiation of dialysis but used intravenous VDRA in March 2015 (OV-IV). Models were adjusted by several factors as follows: Model 1: adjusted for age and gender. Model 2: adjusted for Model 1 plus comorbidity of diabetes, history of cardiovascular disease, BMI, diastolic blood pressure, aortic calcification, eGFR, serum phosphate, serum magnesium, and use of ACEI / ARB. Model 3: adjusted for Model 2 plus serum adjusted calcium. All-cause mortality rates were significantly lower of the Subgroup d than the Subgroup a in model 1 (HR = 0.30, 95% CI = 0.11–0.86, *p* = 0.024). All-cause mortality rates were significantly lower for the Subgroup e than the Subgroup a in model 1 and 2 (HR = 0.43, 95% CI = 0.24–0.77, *p* = 0.004, HR = 0.47, 95% CI = 0.23–0.96, *p* = 0.038, respectively). Details of hazard ratio (CI) and P value of each subgroup in each model were as follows. Significant *p* values are marked with * in the figure. In unadjusted model, b (HR = 0.74, 95% CI = 0.45–1.22, *p* = 0.240); c (HR = 0.77, 95% CI = 0.43–1.40, *p* = 0.396); d (HR = 0.49, 95% CI = 0.28–0.88, *p* = 0.016); e (HR = 0.28, 95% CI = 0.099–0.81, *p* = 0.019). In Model 1, b (HR = 0.82, 95% CI = 0.45–1.49, *p* = 0.520); c (HR = 0.68, 95% CI = 0.41–1.12, *p* = 0.127); d (HR = 0.30, 95% CI = 0.105–0.856, *p* = 0.024); e (HR = 0.43, 95% CI = 0.24–0.768, *p* = 0.004). In Model 2, b (HR = 0.65, 95% CI = 0.30–1.39, *p* = 0.265); c (HR = 0.69, 95% CI = 0.37–1.28, *p* = 0.241); d (HR = 0.37, 95% CI = 0.12–1.105, *p* = 0.075); e (HR = 0.47, 95% CI = 0.23–0.958, *p* = 0.038). In Model 3, b (HR = 0.68, 95% CI = 0.32–1.47, *p* = 0.332); c (HR = 0.74, 95% CI = 0.40–1.38, *p* = 0.337); d (HR = 0.40, 95% CI = 0.13–1.19, *p* = 0.098); e (HR = 0.53, 95% CI = 0.26–1.09, *p* = 0.083). IV: intravenous VDRA group; OV: oral VDRA group; NV: without VDRA group.

Significant differences in cumulative survival rates were observed between the five subgroups (*p* = 0.042; Supplementary Figure 2). The HRs for all-cause mortality in the five subgroups are shown in Figure 4. In Model 1, the all-cause mortality rates of Subgroup d were significantly lower than those of Subgroup a (HR, 0.30;95% CI 0.11–0.86; *p* = 0.02). Furthermore, in Models 1 and 2, the Subgroup e all-cause mortality rates were significantly lower than those of Subgroup a (HR 0.43 [95% CI 0.24–0.77], *p* = 0.004; HR 0.47 [95% CI 0.23–0.96], *p* = 0.038, respectively). However, no significant differences in mortality were observed between Subgroups a and e in Model 3 (HR, 0.53;95% CI 0.26–1.09], *p* = 0.083).

## Discussion

In Japan, the patient survival rate within 5 years after the initiation of dialysis is of significant concern. We focused on the impact of the VDRA dosage form on the short-term survival of incident hemodialysis patients during the introduction period of hemodialysis. This study was unique because the subjects were patients with a relatively short dialysis vintage (<3.5 years). We investigated the short-term survival of patients who were prescribed different VDRA dosage forms and found evidence based on data from real-world clinical settings that supported the effects of VDRA on mortality in the early period after dialysis initiation.

Previous studies have reported that VDRAs can provide several benefits to CKD patients in addition to the classical effects on calcium metabolism. In CKD patients, both OV and IV, or native vitamin D have been suggested to improve survival [27,28], although the exact mechanisms remain under discussion. However, pleiotropic effects on renin-angiotensin system regulation and infectious disease reduction have been reported [8,29,30]. Some studies have also reported that VDRA may prevent left ventricular hypertrophy. On the other hand, paricalcitol capsule benefits in renal failure-induced cardiac morbidity (PRIMO) [31] and the effect of paricalcitol on left ventricular mass and function in CKD (OPERA) [32] studies did not demonstrate that paricalcitol improved left ventricular hypertrophy in pre-dialysis patients; therefore, these effects remain controversial in those patients [10,31–33]. Obi et al. reported that the early use of VDRAs (within at least 2 years after dialysis initiation) was associated with lower infection-related mortality based on data from a Japanese nationwide registry [34]. Although the use of VDRAs for CKD patients remains controversial, VDRAs were used within at least 3.5 years after dialysis initiation in our study, similar to the Japanese nationwide data. According to the decline in kidney function, serum calcitriol levels decrease to below the normal lower limit far earlier than dialysis initiation. Hence, our results suggest that the initiation of VDRA should be considered before the decline in residual kidney function. Although few studies prospectively investigating the association between VDRA or native vitamin D and survival have been published, Shoji et al. reported that OV use for hemodialysis patients without secondary hyperparathyroidism did not improve survival rate in a large-scale randomized controlled trial conducted in Japan [35]. In that study, >900 patients were enrolled, and mortality and CVD events were compared between groups with or without OV use. Compared to our study, the dialysis period was longer for patients enrolled in that study. Although we could not directly conclude that VDRA use improved survival due to the observational design of our study, we did demonstrate that patients who had taken OV before dialysis initiation continued to have a relatively better prognosis than those who had not. Consequently, we believe that it may be better to use VDRA in the pre-dialysis stage.

Researchers are unsure of the optimal method for VDRA administration. Native vitamin D is activated by CYP2R1 and CYP27B1 in the liver and kidneys, respectively [36]. Notably, 25-dihydroxyvitamin D can be converted to calcitriol in different types of cells, including monocytes and parathyroid cells [37]. Moreover, calcitriol acts in a paracrine and autocrine manner. Some studies have shown that locally produced calcitriol may also play an important role in the pleiotropic effects of vitamin D [38,39]. Unlike other countries, it is uncommon to take native vitamin D as a supplement in Japan, and there is no insurance coverage for native vitamin D prescriptions. Therefore, if a physician in Japan intends to prescribe drugs for vitamin D deficiency in a patient undergoing hemodialysis, the options are limited to oral VDRA (oral calcitriol or alpha-calcidol) or intravenous VDRA (intravenous calcitriol or maxacalcitol). Nephrologists involved in the AICOPP were instructed to refer to the JSDT clinical practice guidelines, which recommend that the target ranges for serum phosphate and adjusted calcium concentrations should be between 3.5 and 6.0 mg/dL and 8.4–10.0 mg/dL, respectively. The target range for intact PTH levels was between 60 and 180 pg/mL in the 2006 version [40], and between 60 and 240 pg/mL in the 2012 version [19]. We suspect that clinicians hesitated to prescribe VDRA for patients in the NV group (low serum PTH and alkaline phosphatase [ALP] levels, high serum calcium) because they surmised that bone turnover was low. In contrast, physicians might have expected that patients in the IV group (high serum intact PTH and ALP levels, low serum calcium) did not suffer from irreversible hyperparathyroidism, and therefore were likely to respond to an intravenous VDRA pulse due to their relatively short dialysis vintage. Because the number of patients with diabetes was remarkably low in the IV group, we further calculated the hazard ratios for oral or intravenous VDRA in cases with and without stratified analysis. As a result, the significant difference between NV and IV almost disappeared. However, the IV hazard ratio was relatively low, and we presume that overall there was a greater tendency to prescribe VDRA in the IV group, as described above. Therefore, the lack of significance was likely due to insufficient power in the analysis.

The present study has some limitations. First, the observational design and some inherent biases may have affected the results, even though we performed multivariate analyses. The gap between dialysis initiation and the initiation of follow-up was also a concern. In addition, because the AICOPP study was designed to investigate the association between data at the initiation of hemodialysis and prognosis, we could not obtain exact information on the timing and duration of VDRA treatment initiation and transition to laboratory data. Therefore, we set baseline, not at the initiation of VDRA treatment, but instead at the initiation of hemodialysis, and compared mortality rates between the OV and IV groups (limited to patients who had already been administered VDRAs at dialysis initiation). Second, patient selection bias may have occurred because we included only patients who survived at all three selected points, and due to lack of information we could not exclude patients who had died by the surveillance cutoff in March 2015. Hence, we could not clarify the relationship between VDRA use and mortality in patients with shorter survival periods after dialysis initiation. Third, the criteria for dialysis initiation and rules for the use of VDRA and calcimimetics were undetermined. However, all physicians in the participating facilities who decided to initiate dialysis were nephrologists certified by the Japanese Society of Nephrology. Therefore, we assumed that there were few differences between patients in the timing of dialysis initiation. Unfortunately, we could not survey the use of VDRAs, phosphate binders, and calcimimetics throughout the entire follow-up period, although these medications have been reported to be related to some prognoses [41–44]. We presumed that patients who were not administered VDRAs, both at dialysis initiation and on 1st March 2015, were never administered VDRAs during the entire follow-up period. Finally, we assumed that the number of patients treated with calcimimetics was small because the dialysis vintage was not excessively long.

In conclusion, this study demonstrated the impact of the VDRA dosage form on the short-term survival of incident hemodialysis patients during the hemodialysis introduction period. These results suggest that relatively early initiation of intravenous VDRA in patients who begin hemodialysis may have some clinical potential. Consequently, future prospective, randomized controlled studies maintaining strict management of calcium and phosphate are warranted.

## Supplementary Material

Supplementary Material

## Data Availability

The datasets analyzed during the current study are available from the corresponding author on reasonable request.
