# Supplementary material for: Relationship between selection of dosage forms of vitamin D receptor activators and short-term survival of patients on hemodialysis
Source: Ren Fail. 2021 Nov 17;43(1):1528–38. doi: 10.1080/0886022X.2021.1995423 (PMC8604548; doi:10.1080/0886022X.2021.1995423)
Supplement: Supplementary Material [file IRNF_A_1995423_SM6761.pdf]

**Supplementary material for Koshi-Ito, et al., "Relationship between selection of dosage forms of Vitamin D Receptor Activators and short-term survival of patients on hemodialysis", *Renal Failure*, 2021.**

Supplementary Figure 1

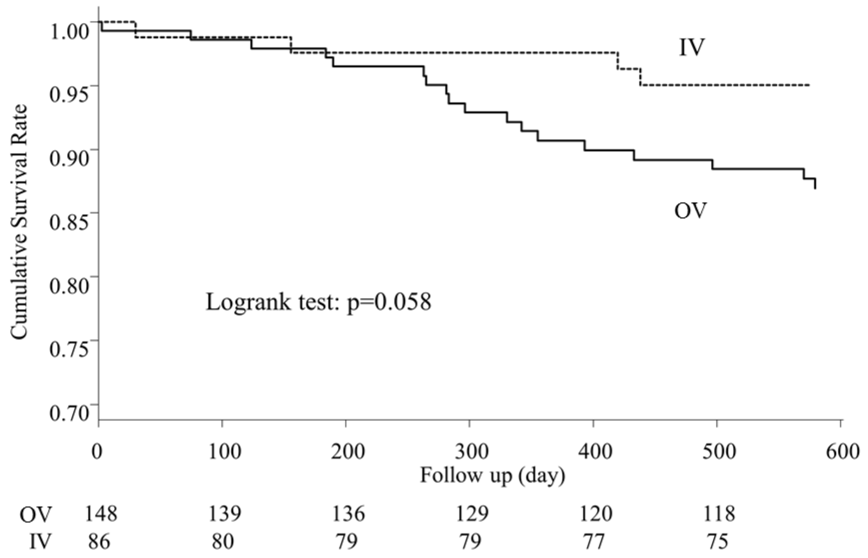


**Supplementary Figure 1.** Kaplan-Meier curve for the cumulative survival between the two subgroups limited to patients who had already taken VDRAs at dialysis initiation.

No significant differences between the cumulative survival rates were observed for the two groups (*p* = 0.058).

IV; intravenous VDRA, OV; oral VDRA, NV; without VDRA

Supplementary Figure 2

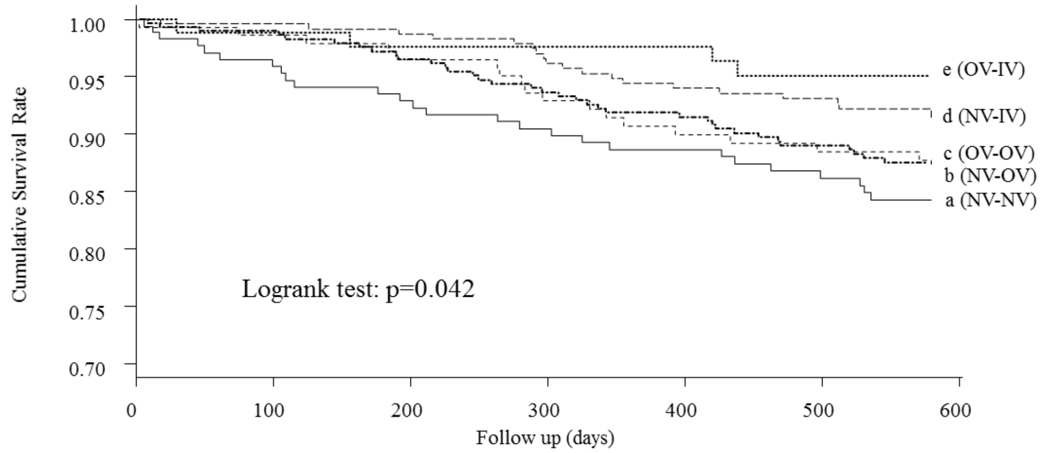


| | | | | | | |
|---|---|---|---|---|---|---|
| a (NV-NV) | 177 | 160 | 153 | 149 | 145 | 138 |
| b (NV-OV) | 299 | 278 | 270 | 262 | 255 | 243 |
| c (OV-OV) | 148 | 139 | 136 | 129 | 120 | 118 |
| d (NV-IV) | 257 | 235 | 231 | 220 | 215 | 211 |
| e (OV-IV) | 86 | 80 | 79 | 79 | 77 | 75 |

**Supplementary Figure 2.** Kaplan-Meier curves for the cumulative survival between the five subgroups.

The main three groups were subdivided into five subgroups according to which form of VDRA was used at both of two points: initiation of dialysis (at baseline) and March 2015 (at interim report). Subgroup a, which did not use VDRA at both points (NV-NV); Subgroup b, which used oral VDRA in March 2015 but not at dialysis initiation (NV-OV); Subgroup c, which used oral VDRA at both points (OV-OV); Subgroup d, which had not used any forms of VDRA at initiation of dialysis but used intravenous VDRA in March 2015 (NV-IV); and Subgroup e, which had used oral VDRA at initiation of dialysis but used intravenous VDRA in March 2015 (OV-IV).
Significant differences between the cumulative survival rates were observed for the five groups ($p$ = 0.042).
IV; intravenous VDRA, OV; oral VDRA, NV; without VDRA
